# Association of cytochrome P450 genetic polymorphisms with neoadjuvant chemotherapy efficacy in breast cancer patients

**DOI:** 10.1186/1471-2350-13-45

**Published:** 2012-06-15

**Authors:** Tatyana A Seredina, Olga B Goreva, Valeria O Talaban, Alevtina Yu Grishanova, Vyacheslav V Lyakhovich

**Affiliations:** 1 Department of Xenobiochemistry, Institute of Molecular Biology and Biophysics of Siberian Branch of the Russian Academy of Medical Sciences (RIMBB SB RAMS), 2, Timakova str, Novosibirsk, 630117, Russia

**Keywords:** Cytochrome P450 genetic polymorphisms, Neoadjuvant chemotherapy efficacy, Breast cancer

## Abstract

**Background:**

The enzymes of the cytochrome P450 family (CYPs) play an important role in the metabolism of a great variety of anticancer agents; therefore, polymorphisms in genes encoding for metabolizing enzymes and drugs transporters can affect drug efficacy and toxicity.

**Methods:**

The genetic polymorphisms of cytochrome P450 were studied in 395 patients with breast cancer by RLFP analysis.

**Results:**

Here, we studied the association of functionally significant variant alleles of CYP3A4, CYP3A5, CYP2B6, CYP2C8, CYP2C9 and CYP2C19 with the clinical response to neoadjuvant chemotherapy in breast cancer patients. A significant correlation was observed between the CYP2C9*2 polymorphism and chemotherapy resistance (OR = 4.64; CI 95% = 1.01 – 20.91), as well as between CYP2C9*2 heterozygotes and chemotherapy resistance in women with nodal forms of breast cancer and a cancer hereditary load (OR = 15.50; CI 95% = 1.08 – 826.12) when the potential combined effects were examined. No significant association between chemotherapy resistance and the other examined genotypes and the potential combined clinical and tumour-related parameters were discovered.

**Conclusion:**

In conclusion, CYP2C9*2 was associated with neoadjuvant chemotherapy resistance (OR = 4.64; CI 95% = 1.01 – 20.91) in the population of interest.

## Background

Individual variability in drug efficacy and toxicity resulting in different clinical responses is common in therapeutic areas, including breast cancer. It is an important problem in clinical practice because it can lead to therapeutic failure and adverse effects. A wide variety of factors may influence drug availability and drug response, such as race, sex, diet, differences in drug pharmacokinetics and pharmacodynamics, etc. However, the importance of all of these factors is secondary to the effect of polymorphisms in drug metabolizing enzymes, drug transporters and drug targets. Polymorphisms in the genes encoding enzymes responsible for the metabolism of drugs and other xenobiotics and the functional significance of these polymorphisms are critical for predicting clinical outcomes [1-3].

The members of the cytochrome P450 superfamily are involved in phase I of the xenobiotic metabolizing process. These enzymes catalyze the oxidation of many exogenous and endogenous compounds and are responsible for the metabolism of approximately 90% of clinically prescribed drugs. The CYPs are known to be involved in the metabolism of many anticancer drugs, including cyclophosphamide, 5-fluorouracil, adreamicin, xeloda, ifosfamide, etoposide, paclitaxel, etc. [4]. It was recently shown that the prodrug cyclophosphamide is activated by CYP2B6, CYP2C9 and CYP2C19 and is inactivated by CYP3A4 and CYP3A5 [5]. Xeloda is metabolized by CYP2B6, CYP2C8 and CYP2C9, while adreamicin and methatrexane are metabolized by CYP3A4 [4,6].

Polymorphisms in genes encoding for metabolizing enzymes and drug transporters can affect drug efficacy and toxicity. *CYP2C8* and *CYP2C9* are considered highly variable genes and have more than 14 and 34 polymorphic alleles, respectively (http://www.imm.ki.se/CYPalleles). Most of the *CYP2C9* polymorphisms are known to decrease the enzymatic activity of the enzyme. The CYP2C8*3CYP2C9*2 and CYP2C9*3 polymorphic alleles frequently occur among Caucasians and lead to non-synonymous mutations, which result in decreased activity of CYP2C8 and CYP2C9 [7]. The CYP2C8 enzyme is involved in the metabolism of cyclophosphamide, ifosphamide and paclitaxel, while CYP2C9 metabolizes cyclofosphamide, ifosphamide and tamoxifen and activates tegafur [5,8].

At least 28 CYP2C19 variant alleles have been previously described (http://www.imm.ki.se/CYPalleles), 9 of which encode for inactive enzymes. Mutations in exon 5 (CYP2C19*2) and exon 4 (CYP2C19*3) are the most common polymorphisms. Both CYP2C19*2, which causes a 40-nucleotide deletion and a frameshift, and CYP2C19*3, which leads to a premature stop codon, result in the production of a truncated protein without enzymatic activity [9]. CYP2C19 plays a role in the metabolism of the anticancer drugs cyclofosphamide, ifosphamide, tamoxifen and thalidomide [8].

CYP2B6 may have as many as 29 polymorphisms, but only CYP2B6*5 and CYP2B6*7 are likely to be functionally significant and result in decreased enzymatic activity [10,11]. CYP2B6 is involved in the activation of anticancer drugs such as cyclophosphamide and ifosphamide [5].

CYP3A is the P450 cytochrome with the widest range of drug substrates. For the majority of people, it is also the most abundant cytochrome in the liver. CYP3A4 activity has a wide range of individual activity, up to a 40-fold difference, which may be related to the individual’s health status, environment, hormones or genetics. Over 30 CYP3A4 alleles have been described, including 18 associated, non-synonymous mutations (http://www.imm.ki.se/CYPalleles). The most common allele is CYP3A4*2, which is found in Caucasians and encodes for a protein with decreased activity. A number of upstream polymorphisms have also been detected. The most extensively studied of these is CYP3A4*1B. This polymorphism alters the putative transcriptional regulatory element - nifidipine oxidase specific element (NFSE), which is known to be required for the expression of CYP3A4 [12]. Although the CYP3A4*1B allele was initially shown to result in a 1.5-fold increase in transcription in vitro, subsequent reports have indicated no change in its enzymatic activity. Currently, over 11 different *CYP3A5* alleles have been identified. Individuals homozygous for the CYP3A5*3 allele appear to not express a functional version of the CYP3A5 enzyme due to a cryptic splice site that results in the incorporation of intronic sequence in the mature mRNA and the production of a truncated protein due to a premature appearance of termination codon [13]. This is important in anticancer therapies as CYP3A is involved in the metabolism of many drugs, including cyclophpsphamide, ifosfamide, docetaxel, paclitaxel, etoposide, irinotecan, tamoxifen, imatenib, gefenitib and vinca-alkaloids [4].

We studied the association of the functionally significant variant alleles of *CYP3A4*, *CYP3A5*, *CYP2B6*, *CYP2C8*, *CYP2C9* and *CYP2C19* with the clinical response to neoadjuvant chemotherapy in breast cancer patients.

## Methods

### Patients

From 1991 to 2007, 395 women (mean age of 52.00 ± 9.89) with a morphologically confirmed diagnosis of breast cancer at stages T_1-4_ N_0-3_ M_0_ were observed at the Tomsk Cancer Research Institute in the Tomsk Scientific Center of the RAMS. Of these, 261 were treated with one of the following neoadjuvant chemotherapies: CMF or CMXeloda (Cyclophosphamide, Methotrexate and either Fluorouracil or Xeloda), FAC (Fluorouracil, Adreamicin and Cyclophosphamide) and CAF or CAXeloda (Cyclophosphamide, Adreamicin and either Fluorouracil or Xeloda). The chemotherapeutic effect was estimated after 2–4 chemotherapy courses through ultrasonic scanning and mammography according to the World Health Organization criteria. Complete remission (CR) was observed when no breast tumour; partial remission (PR) when the reduction in the tumour area was 50-100%; and stable disease (SD) when the tumour area was reduced 0–50, progressive disease (PD) was recorded if the tumour area increased or if a new lesion was detected. Complete remission was achieved in 3% of the patients; the partial remission rate was 47%; the stable disease rate was 46% and 4% of the patients showed progression of the disease. The patients were divided into two groups according to the results of the neoadjuvant therapy; patients classified as having CR, PR or SD formed the positive response group, while patients classified as showing PD made up the negative chemotherapy response group. The local ethical review boards approved the study protocol (Protocol N3 from 08.06.2005), and all patients provided written, informed consent before acceptance into the study in accordance with the Declaration of Helsinki.

### Genotyping

The genetic polymorphisms of cytochrome P450 were studied in 395 patients with breast cancer by RLFP analysis. Genomic DNA was kindly provided by the Tomsk Oncology Center SB RAMS. Oligonucleotide primers for the CYP2B6*5CYP2C8*2CYP2C8*3CYP2C19*2 and CYP2C19*3 polymorphisms and restriction endonucleases were designed using the program Vector NTI 8.0. The primers for the CYP2C9*2CYP2C9*3CYP3A4*2CYP3A4*B and CYP3A5*3 polymorphisms were described earlier [14-17]. All of the primer sequences are provided in Table 1.

**Table 1:** Primers and restriction endonucleases for the CYP450 polymorphisms genotyping

**Polymorphism**	**Oligonucleotide primers**	**Amplicone**	**Restriction endonucleases**	Restriction products, b.p.*
CYP3A5*3 (intron 3)	f 5'-catcagttagtagacagatga-3'	293	Ssp I	148 125 20
	r 5'-ggtccaaacagggaagaaata-3'			(168 125)
CYP3A4*1B (5'-NTR)	f 5'-tccaggcataggtaaagatc-3'	111	Acc 36 I	111
	r 5'-aatctattaaatcgcctctcac-3'			(85 26)
CYP3A4*2 (exon 6)	f 5'-ttttttggatccattctttgtc-3'	124	Bst MA I	98 26
	r 5'-ttttaagtggatgaattacatggt-3'			(124)
CYP2C9*2 (exon 3)	f 5'-cactggctgaaagagctaacagag-3'	372	Asp S9 I	179 119 74
	r 5'-gtgatatggagtagggtcacccac-3'			(253 119)
CYP2C9*3 (exon 7)	f 5'-aggaagagattgaacgtgtga-3'	130	ERh I	130
	r 5'-ggcaggctggtggggagaaggccaa-3'			(104 26)
CYP2C19*2 (exon 5)	f 5'-ccagagcttggcatattgta-3'	230	Sma I	109 121
	r 5'-gaagcaatcaataaagtcccga-3'			(230)
CYP2C19*3 (exon 4)	f 5'-ctgggctgtgctccct-3'	147	BamH I	128 19
	r 5'-acttggccttacctggct-3'			(147)
CYB2B6*5 (exon 9)	f 5'-aatacccccaacataccacatc-3'	121	Bst F5 I	105 16
	r 5'-gcggggagtcagagccatt-3'			(121)
CYP2C8*2 (exon 5)	f 5’-aaagtaaaagaacaccaagc-3’	167	Kzo9 I	69 65 33
	r 5’- aaacatccttagtaaattaca-3’			(98 69)
CYP2C8*3 (exon 3/8)	f 5’- aggcaattccccaatatctc-3’	467	BseR I	310 111 46
	r 5’-caggatgcgcaatgaagac-3’			(356 111)

* - fragments of the wild type alleles, fragments of the mutant type alleles parentheses.

The PCR reactions were carried out in a 20 μl volume and contained 1x PCR buffer, 1–2 mM MgCl_2_, 250 μM dNTPs, 0.5 μM primers, 2 U Taq DNA polymerase (Medigen, Russia) and 30 ng of genomic DNA. Thermal cycling was performed using an initial denaturation time of 3 min at 94°C followed by 33 cycles of 15 s at 94°C, 30 s at 55°C and 30 s at 72°C. A terminal extension time of 5 min at 72°C was used. The reaction products were digested with the appropriate restriction endonucleases (SibEnzyme Ltd., Russia) (Table 1) for 6 hour at 37°C. The fragments were resolved by PAGE on 10% TBE gels and were subsequently stained with ethidium bromide (10 mg/ml) to visualize the bands using the “VersaDoc System” (Bio-Rad, USA).

### Statistical analysis

The allelic and genotype frequencies for all examined genes were calculated for descriptive purposes, and correlation analyses were performed to investigate the relationship between the genotypes and the neoadjuvant chemotherapy outcome. The data were analyzed using EpiInfo 6.0. Differences in the distributions of the variables were analyzed by the *χ*^2^ or Fisher’s exact tests, where the cell numbers were less than 5. We also calculated the odds ratios (OR), 95% confidence intervals (CI) and the levels of significance. A *p* value less than or equal to 0.05 was considered statistically significant. All of the genotype distributions were analyzed for Hardy-Weinberg equilibrium.

## Results and discussion

### The distribution of the cytochrome P450 genotypes and allele frequencies in the breast cancer patients

The cytochrome P450 genetic polymorphisms CYP2C8*2, CYP2C8*3, CYP2C9*2, CYP2C9*3, CYP2C19*2, CYP2C19*3, CYP3A4*1B, CYP3A4*2, CYP3A5*3 and CYP2B6*5 were investigated in 395 female breast cancer patients. Table 2 shows the distribution of the cytochrome P450 genotypes and allele frequencies in the patients. The distribution of the genotype frequencies in breast cancer patients is in agreement with the expected frequencies for the majority of the investigated polymorphisms. Significant deviation from Hardy-Weinberg equilibrium was observed for CYP2C19*2 (p = 0.003) and CYP2B6*5 (p = 0.0008). This may be indicative of the functional significance of this locus or the variants being in non-equilibrium, linking this locus to breast cancer.

**Table 2:** Genotype and allele frequencies of the CYP P450 genes in breast cancer patients

**Nomenclature**	**rs**	**Nucleotide changes**	**Effect**	**n**	**Genotype frequencies, %**			**Allele frequencies, %**	
					**wt**	**ht**	**vt**	**p**	**q**
CYP2C8*2	rs11572103	T805A	I269F	383	99.22	0.78	0.00	99.61	0.39
CYP2C8*3	rs11572080+ rs10509681	G416A+ A1196G	R139K + K399R	390	83.85	15.64	0.51	91.67	8.33
CYP2C9*2	rs1799853	C430T	R144C	391	79.54	20.20	0.26	89.64	10.36
CYP2C9*3	rs1057910	A1075C	I359L	394	81.22	17.77	1.02	90.10	9.90
CYP2C19*2	rs4244285	G681A	Splicing defect	393	58.52	39.19	2.29	78.12	21.88
CYP2C19*3	rs4986893	G636A	W212X	388	97.94	2.06	0.00	98.97	1.03
CYP3A4*1B	rs2740574	A-392 G	5'NTO change	389	94.86	5.14	0.00	97.43	2.57
CYP3A4*2	rs55785340	T664C	S222P	391	100.00	0.00	0.00	100.00	0.00
CYP3A5*3	rs776746	A6986G	R487C	392	0.26	11.99	87.76	6.25	93.75
CYP2B6*5	rs3211371	C1459T	Splicing defect	390	85.64	12.56	1.79	91.92	8.08

The frequencies of the CYP2C8*2 and CYP2C8*3 mutant alleles were 0.39% and 8.33% in the investigated group of breast cancer patients. According to the literature, the CYP2C8*2 allele is known to be found only in African Americans, which carry the allele at a frequency of 18%, while the CYP2C8*3 mutant allele occurs primarily in Caucasians at a frequency of 13%. Neither allele has so far been identified in Asians [18,19].

In this group of breast cancer patients the frequency of the CYP2C9*2 and CYP2C9*3 mutant alleles was 10.36% and 9.90%, which was not significantly different from the number found in the literature for Caucasians, i.e., 8-19% and 0–8.5% [1,7,20-23] for CYP2C9*2 and CYP2C9*3, respectively, but it is significantly higher than that found in African Americans, who carry the alleles at a frequency of 1% and 0.5%, respectively. The CYP2C9*2 allele has not been detected in a Chinese population, and the frequency of the CYP2C9*3 allele was 2–2.6% [22,24]. The CYP2C19*2 mutant allele was found at a frequency of 21.9% in this study, which is not significantly different from previous reports in the literature for Caucasians (15 20%) [1,7] and Japanese (26.7%) [25]. The CYP2C19*3 mutant allele frequency was 1.03% in our group of breast cancer patients, which was not different from the value found in the literature data for Caucasians (0.0%) or Asians (3.75%) [26]. This allele occurs only in heterozygocity. None of the investigated patients were homozygotes for the CYP2C19*3 allele. The CYP2B6*5 mutant allele frequency was 8.1%, which was not significantly lower than the published value for Caucasians (9.1%) and higher than that published for Asians (4.2%).

The frequency of the CYP3A4*1B mutant allele was 2.57% in this group of breast cancer patients; the literature reports significant interethnic variations in this allele (2-9% for Caucasians, 35-66% for African Americans and 0% for Taiwanese and Chinese [27]). The CYP3A4*2 mutant allele was not present in the investigated group of breast cancer patients, while in its frequency in Caucasians ranges from 1.1% in a German population to 4.5% in a Portuguese population [28].

The frequency of the CYP3A5*3 polymorphic allele in the total population sampled was 93.75%. This agrees with the previously published data, which reports a total frequency of the CYP3A5*3 allele of 91.7- 94.2% in Caucasians [14,27,29] and 66.7-75% in Asians [30].

### The cytochrome P450 polymorphisms and the chemotherapy efficacy in the breast cancer patients

Table 3 shows the distribution of the polymorphic variants of the analyzed genes in the breast cancer patients along with the known efficacy of the neoadjuvant chemotherapy for the CYP2 family. The study of the association of the CYP2B6*5, CYP2C8*2, CYP2C8*3, CYP2C9*2, CYP2C9*3, CYP2C19*2 and CYP2C19*3 variants with a negative response to neoadjuvant chemotherapy in breast cancer patients included an odds ratio evaluation indicating the probability of poor chemotherapeutic efficacy in individuals with certain genotypes. The observed distribution of the most common genotypes in breast cancer patients are in Hardy-Weinberg equilibrium with the exception of CYP2B6*5 (p = 0.0198) and CYP19*2 (p = 0.0003). The enrichment of the CYP2B6*5 and CYP19*2 alleles in this group may be due to the population possessing its own pool of alleles, resulting in different frequencies of unfavorable alleles. The risk of an insufficient response to breast cancer neoadjuvant chemotherapy in CYP2C9*2 heterozygotes was 4.64-fold higher (OR = 4.64, p = 0.02) than in patients with the wild type allele. The impaired efficacy of neoadjuvant chemotherapy in patients containing the CYP2C9*2 mutant allele might be a result of the enzymatic activity of CYP2C9, which is involved into the chain reaction responsible for the conversion of the cyclophpsphamide prodrug into an active metabolite [29,30].

**Table 3:** Distribution of CYP2 genotypes and risk of neoadjuvant chemotherapy resistance developing in breast cancer patients

**Polymorphism**	**Genotype**	**Positive neoadjuvant chemotherapy response**		**Negative neoadjuvant chemotherapy response**		**OR**	**CI (95%)**	**p**
		**n**	**%**	**n**	**%**			
CYP2B6*5 C1459T	**CC**	**205**	82.66	**9**	90.00	1.89	0.25 - 84.64	0.99
	**CT**	**37**	14.92	**1**	10.00	1.13	0.54 - 2.40	0.99
	**TT**	**6**	2.42	**0**	0.00	0.63	0.01 - 4.82	0.99
CYP2C8*2 T805A	**TT**	**243**	99.59	**10**	100.00	0.04	0.01 - 3.55	0.08
	**TA**	**1**	0.41	**0**	0.00	24.30	0.28 - 1914.18	0.08
	**AA**	**0**	0.00	**0**	0.00	*		
CYP2C8*3 G416A + A1196G	**GG**	**203**	82.19	**6**	60.00	0.33	0.07 - 1.64	0.09
	**GA**	**44**	17.81	**4**	40.00	3.08	0.61 - 13.53	0.09
	**AA**	**0**	0.00	**0**	0.00	*		
CYP2C9*2 C430T	**CC**	**204**	82.26	**5**	50.00	**0.22**	0.05 - 0.99	**0.02**
	**CT**	**44**	17.74	**5**	50.00	**4.64**	1.01 - 20.91	**0.02**
	**TT**	**0**	0.00	**0**	0.00	*		
CYP2C9*3 A1075C	**AA**	**211**	84.40	**8**	80.00	0.74	0.14 - 7.41	0.66
	**AC**	**36**	14.40	**1**	10.00	0.66	0.01 - 5.03	0.99
	**CC**	**3**	1.20	**1**	10.00	9.15	0.16 - 124.95	0.15
CYP2C19*2 G681A	**GG**	**136**	54.62	**8**	66.67	3.32	0.64 - 32.61	0.19
	**GA**	**109**	43.78	**2**	16.67	0.32	0.03 - 1.66	0.20
	**AA**	**4**	1.61	**2**	16.67	6.13	0.11 - 69.01	0.20
CYP2C19*3 G636A	**GG**	**241**	97.97	**10**	100.00	0.21	0.02 - 10.78	0.23
	**GA**	**5**	2.03	**0**	0.00	4.82	0.09 - 48.97	0.23
	**AA**	**0**	0.00	**0**	0.00	*		

*- OR, CI (95%) and p-values were not applicable to these samples.

The risk of poor neoadjuvant chemotherapy efficacy is much higher in CYP2C8*2 heterozygotes (OR *=* 24.30) and slightly higher in CYP2C8*3 heterozygotes (OR = 3.08) at a level of statistical significance close to the proposed level (p = 0.08 and p = 0.09, respectively, versus p < 0.05). Furthermore, the CYP2C9*3 (OR = 9.15) and CYP2C19*2 (OR = 6.13) mutant type genotypes and heterozygotic CYP2C19*3 (OR = 4.82) genotype are associated with a low efficacy of neoadjuvant chemotherapy; however, these associations were not found to be statistically significant (p > 0.05). It appears that the CYP2B6*5, CYP2C8*2, CYP2C8*3, CYP2C9*3, CYP2C19*2 and CYP2C19*3 mutant alleles are not a factor in resistance to neoadjuvant chemotherapies in breast cancer patients.

Table 4 shows the distribution of the polymorphic variants of the genes studied here in breast cancer patients along with the known efficacy of the neoadjuvant chemotherapy for the CYP3 family. Gene polymorphisms association with neoadjuvant chemotherapy efficacy were analyzed for the CYP3A4*1B and CYP3A5*3 polymorphisms. CYP3A4*2 was not included in the study because no heterozygotes or homozygotes containing the CYP3A4*2 mutant allele were found in the population. For CYP3A4*1B heterozygotes, the risk of a negative response to neoadjuvant chemotherapy is 24.9-fold higher than in carriers of the wild type allele, which has a level of significance (p = 0.08) that approaches the significance threshold (p = 0.05). No significant association between CYP3A5*3 genotypes and chemotherapy resistance were discovered.

**Table 4:** Distribution of CYP3 genotypes and risk of neoadjuvant chemotherapy resistance developing in breast cancer patients

**Polymorphism**	**Genotype**	**Positive neoadjuvant chemotherapy response**		**Negative neoadjuvant chemotherapy response**		**OR**	**CI (95%)**	**p**
		**n**	**%**	**n**	**%**			
CYP3A4*1B A-392 G	**AA**	**234**	94.74	**9**	90.00	0.04	0.01 - 3.47	0.08
	**AG**	**13**	5.26	**1**	10.00	24.90	0.29 - 1961.25	0.08
	**GG**	**0**	0.00	**0**	0.00	*		
CYP3A4*2 T664C	**TT**	**249**	100.00	**10**	100.00	*		
	**TC**	**0**	0.00	**0**	0.00	*		
	**CC**	**0**	0.00	**0**	0.00	*		
CYP3A5*3 A6986G	**AA**	**0**	0.00	**0**	0.00	*		
	**AG**	**33**	13.31	**1**	10.00	0.72	0.02 - 5.53	0.99
	**GG**	**215**	86.69	**9**	90.00	1.38	0.18 - 62.36	0.99

*- OR, CI (95%) and p-values were not applicable to these samples.

The analyses performed here cover the distribution of cytochrome genetic polymorphisms and their association with the known efficacy of neoadjuvant chemotherapy in breast cancer patients known to have a cancer hereditary load (cancer cases in patient’s relatives), a clinical form of cancer, degree of malignancy and histological type of tumour. In CYP2C9*2 heterozygotes with a high hereditary load, the risk of tumour resistance to neoadjuvant chemotherapy was 7.6-fold higher than wild type homozygotes (OR = 7.6, p = 0.04) (Table 5). A similar association was observed in patients with the nodal form of breast cancer. The risk of tumour resistance to chemotherapy for CYP2C9*2 heterozygotes was 6.83-fold higher than in wild type homozygotes (Table 5; OR = 6.83, p = 0.019). In CYP2C9*2 heterozygotes with nodal form of cancer and a cancer hereditary load, the risk of resistance to chemotherapy was 15.5-fold higher than those with the wild type genotype (OR = 15.5, p = 0.02). Patients with other clinical features and genetic variants of the investigated cytochromes showed no statistically important association with neoadjuvant chemotherapy efficacy.

**Table 5:** Risk of chemotherapy resistance in breast cancer patients with different CYP2C9*2 genotypes and tumour characteristics

**Group**	Allele/ Genotype CYP2C9*2 C430T	**Positive neoadjuvant chemotherapy response**		**Negative neoadjuvant chemotherapy response**		**OR**	**CI (95%)**	**p**
		**n**	**%**	**n**	**%**			
**Cancer hereditary load**	**C**	**167**	91.76	**7**	70.00	4.77	0.71 - 23.47	
	**T**	**15**	8.24	**3**	30.00			
	**CC**	**76**	83.52	**2**	40.00	0.13	0.01 - 1.29	**0.04**
	**CT**	**15**	16.48	**3**	60.00	7.60	0.78 - 95.24	**0.04**
	**TT**	**0**	0.00	**0**	0.00	*		
**Nodal form**	**C**	**371**	91.83	**10**	71.43	4.50	0.97 - 16.60	
	**T**	**33**	8.17	**4**	28.57			
	**CC**	**169**	83.66	**3**	42.86	0.15	0.02 - 0.92	**0.019**
	**CT**	**33**	16.34	**4**	57.14	6.83	1.09 - 48.12	**0.019**
	**TT**	**0**	0.00	**0**	0.00	*		
**Cancer hereditary load + nodal form**	**C**	**136**	91.89	**3**	50.00	11.33	1.32 - 91.23	
	**T**	**12**	8.11	**3**	50.00			
	**CC**	**62**	83.78	**0**	0.00	0.06	0.01 - 0.91	**0.02**
	**CT**	**12**	16.22	**3**	100.00	15.50	1.08 - 826.12	**0.02**
	**TT**	**0**	0.00	**0**	0.00	*		

*- OR, CI(95%) and p-values were not applicable to these samples.

## Conclusions

CYP2C9*2 polymorphism is associated with neoadjuvant chemotherapy efficacy in breast cancer patients (OR = 4.64; CI 95% = 1.01 – 20.91).

## Competing interests

The authors declare that they have no competing interests.

## Authors' contributions

TAS, OBG, VOT carried out the molecular genetic studies, performed the statistical analysis and drafted the manuscript. AYuG and VVL conceived of the study, and participated in its design and coordination and conducted data acquisition. All authors read and approved the final manuscript.

## Pre-publication history

The pre-publication history for this paper can be accessed here:

http://www.biomedcentral.com/1471-2350/13/45/prepub
